# Secondary Structure of Human *De Novo* Evolved Gene Product NCYM Analyzed by Vacuum-Ultraviolet Circular Dichroism

**DOI:** 10.3389/fonc.2021.688852

**Published:** 2021-08-23

**Authors:** Tatsuhito Matsuo, Kazuma Nakatani, Taiki Setoguchi, Koichi Matsuo, Taro Tamada, Yusuke Suenaga

**Affiliations:** ^1^Institute for Quantum Life Science, National Institutes for Quantum and Radiological Science and Technology, Ibaraki, Japan; ^2^Department of Molecular Carcinogenesis, Chiba Cancer Center Research Institute, Chiba, Japan; ^3^Graduate School of Medical and Pharmaceutical Sciences, Chiba University, Chiba, Japan; ^4^Innovative Medicine CHIBA Doctoral World-leading Innovative & Smart Education (WISE) Program, Chiba University, Chiba, Japan; ^5^Department of Neurosurgery, Chiba Cancer Center, Chiba, Japan; ^6^Hiroshima Synchrotron Radiation Center, Hiroshima University, Hiroshima, Japan

**Keywords:** NCYM, MYCN, de novo evolved protein, secondary structure, VUVCD, perdeuterated protein, SNP, Myc-nick

## Abstract

*NCYM*, a *cis*-antisense gene of *MYCN*, encodes a Homininae-specific protein that promotes the aggressiveness of human tumors. Newly evolved genes from non-genic regions are known as *de novo* genes, and *NCYM* was the first *de novo* gene whose oncogenic functions were validated *in vivo*. Targeting NCYM using drugs is a potential strategy for cancer therapy; however, the NCYM structure must be determined before drug design. In this study, we employed vacuum-ultraviolet circular dichroism to evaluate the secondary structure of NCYM. The SUMO-tagged NCYM and the isolated SUMO tag in both hydrogenated and perdeuterated forms were synthesized and purified in a cell-free *in vitro* system, and vacuum-ultraviolet circular dichroism spectra were measured. Significant differences between the tagged NCYM and the isolated tag were evident in the wavelength range of 190–240 nm. The circular dichroism spectral data combined with a neural network system enabled to predict the secondary structure of NCYM at the amino acid level. The 129-residue tag consists of α-helices (approximately 14%) and β-strands (approximately 29%), which corresponded to the values calculated from the atomic structure of the tag. The 238-residue tagged NCYM contained approximately 17% α-helices and 27% β-strands. The location of the secondary structure predicted using the neural network revealed that these secondary structures were enriched in the Homininae-specific region of NCYM. Deuteration of NCYM altered the secondary structure at D90 from an α-helix to another structure other than α-helix and β-strand although this change was within the experimental error range. All four nonsynonymous single-nucleotide polymorphisms (SNPs) in human populations were in this region, and the amino acid alteration in SNP N52S enhanced Myc-nick production. The D90N mutation in NCYM promoted NCYM-mediated MYCN stabilization. Our results reveal the secondary structure of NCYM and demonstrated that the Homininae-specific domain of NCYM is responsible for MYCN stabilization.

## Introduction

*NCYM* is a *cis*-antisense gene of *MYCN* (1) and encodes an oncogenic protein that promotes the aggressiveness of neuroblastomas (1–5). NCYM regulates the proliferation, invasion, migration, stemness, and apoptosis of cancer cells by stabilizing MYCN (1–5) and/or β-catenin (1, 6) by inhibiting GSK3β. The open reading frame (ORF) is located in the *MYCN* promoter, and mutations introduced during the evolution of Homininae resulted in the generation of the coding transcript of *NCYM* from the non-genic region (1, 5). New genes originating from non-genic regions are known as *de novo* genes (7–10), and NCYM is the first human *de novo* gene product whose oncogenic functions have been validated *in vivo* (5, 9). Because of its *de novo* emergence, NCYM does not shows homology to other known proteins, and its functional domain structure remains unclear.

Newly evolved proteins, including *de novo* gene products (hereinafter *de novo* evolved proteins), are predicted to be small and disordered proteins (11); generally, these high-dimensional structures are difficult to analyze by crystallization/cryo-electron microscopy. Bungard et al. (12) showed that the yeast *de novo* evolved protein Bsc4 folds to a partially ordered three-dimensional structure, forming compact oligomers with high β-sheet content and a hydrophobic core using near-UV circular dichroism as well as nuclear magnetic resonance. They revealed that *de novo* evolved proteins could have some structural order as well as native-like properties; however, the precise locations of the ordered secondary structure in Bsc4 remain unclear.

In this study, we investigated the secondary structure of NCYM by synchrotron radiation vacuum-ultraviolet circular dichroism technology (VUVCD) combined with a neural network. Synchrotron radiation VUVCD enables the analysis of the content and number of segments in the secondary structure of proteins at a wider range of wavelengths compared to near-UV circular dichroism (13, 14). Furthermore, the analysis of results combined with a neural network can predict the locations of the secondary structure of proteins at the amino acid sequence level (15). We carried out VUVCD measurements on both hydrogenated NCYM and perdeuterated NCYM, because some perdeuterated proteins have been reported to change their local structure and to have decreased protein stability compared with their hydrogenated counterparts, affecting their function/activity (16–18). A comparison of the possible differences in the secondary structures between these molecules may provide insights into regions that contribute to molecular stability and function. In addition, we determined whether perdeuterated proteins are helpful for gaining insights into the *de novo* evolved protein structure-function relationship.

## Methods

### Purification of the NCYM Protein by *In Vitro* Cell-Free System

We purchased the following proteins in solution produced by an *in vitro* cell-free system from Taiyo Nippon Sanso Corporation (Tokyo, Japan)

-SUMO-tagged NCYM protein in hydrogenated form at 1.1 mg/mL
-SUMO-tag in hydrogenated form at 0.6 mg/mL
-SUMO-tagged NCYM protein in perdeuterated form at 1.4 mg/mL
-SUMO-tag in perdeuterated form at 1.4 mg/mL

**Figure 1:**
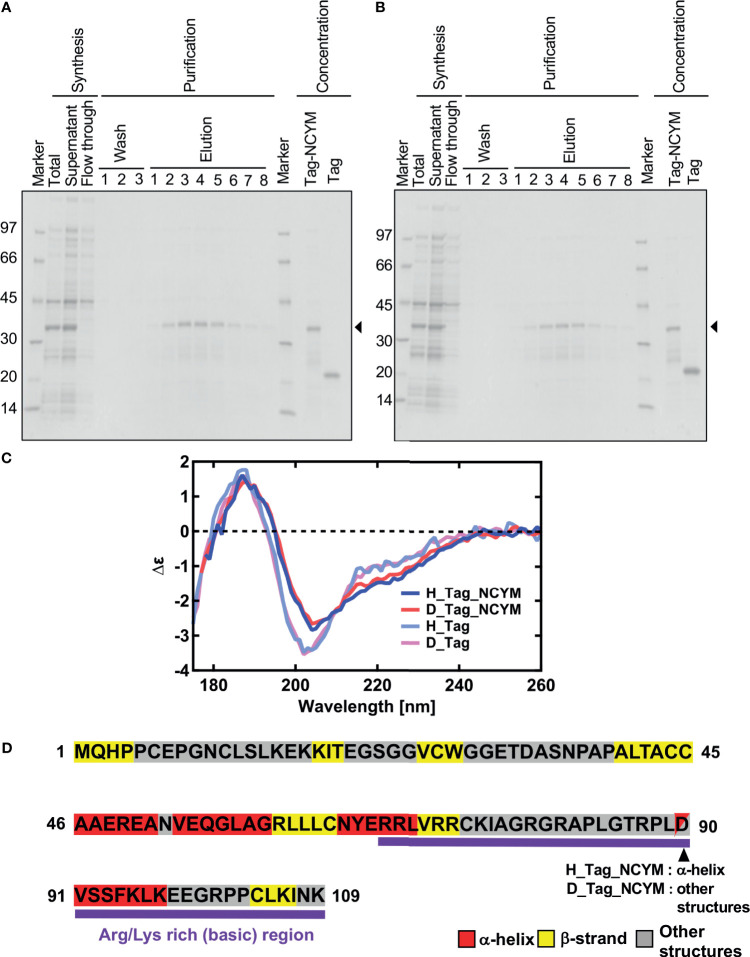
Vacuum-ultraviolet circular dichroism (VUVCD) analyses revealed the secondary structure of NCYM. **(A)** NCYM with SUMO tag (arrow) and the isolated SUMO tag were synthesized and purified using an *in vitro* cell-free system. **(B)** Perdeuterated NCYM with SUMO tag (arrow) and the isolated SUMO tag were synthesized and purified using an *in vitro* cell-free system. **(C)** VUVCD spectra for hydrogenated SUMO-tagged NCYM (H_Tag_NCYM), perdeuterated SUMO-tagged NCYM (D_Tag_NCYM), hydrogenated SUMO tag (H_Tag), and perdeuterated SUMO tag (D_Tag). **(D)** Secondary structure of NCYM predicted using the neural network. Secondary structures are highlighted in red, yellow, and gray for α-helix, β-strand, and other structures, respectively. Arg/Lys-rich (basic) region is highlighted in purple.

The protein concentration was determined spectrophotometrically using the extinction coefficient $E_{280}^{1\%}$ of 3.74 and 1.99 for SUMO-tagged NCYM and the isolated SUMO-tag, respectively. The buffer composition was 20 mM phosphate buffer (pH or pD 8.0), 3 mM DTT. The isolated SUMO tag was separately synthesized and purified from the SUMO-tagged NCYM (**Figures 1A, B**).

### Measurements of VUVCD Spectra

A VUVCD spectrophotometer (Hiroshima Synchrotron Radiation Center, Hiroshima University, Japan) and an assembled-type optical cell with CaF_2_ windows were used to measure the VUVCD spectra of the four samples described above from 260 to 175 nm at 25°C. The isolated SUMO tags were measured for comparison. The details of the optical systems of the spectrophotometer and design of the sample cell have been described previously (19). The path length in the optical cell was adjusted to 50 μm using a Teflon spacer. All spectra were measured under the following conditions: slit, 1.0 mm; time constant, 4 s; scan speed, 20 nm/min; and accumulations, 4–8. The molar circular dichroism, *Δε*, which is in normalized units of CD, was obtained from the path length of the optical cell and solute concentrations. The values of error in the CD spectrum were within 5%, which was mainly attributable to noise and inaccuracy in the optical path length.

### Analysis of the Secondary-Structure Content and Segments of NCYM Using VUVCD and SELCON 3

The contents of α-helices, β-strands, turns, and unordered structures of proteins were estimated from the corresponding VUVCD spectra using the SELCON3 program and a database of VUVCD spectra and secondary-structure contents for 31 reference proteins (13–15, 19). The number of α-helix and β-strand segments was calculated from the distorted α-helix and distorted β-strand contents, respectively (19). The root-mean-square deviation (*δ*) and the Pearson correlation coefficient (*r*) between the X-ray and VUVCD estimates of the secondary-structure contents of the reference proteins were 0.058 and 0.85, respectively (13, 15).

### Analysis of the Positions of the Secondary Structures of NCYM Using VUVCD and Neural-Network Method

The positions of α-helix and β-strand segments in the amino-acid sequence were predicted using a neural-network (NN) method based on the secondary-structure contents and the number of segments obtained in the VUVCD analysis (VUVCD-NN method). The computational protocol is described in detail elsewhere (14). Briefly, we utilized an NN algorithm (20) that predicts the position of secondary structures using the evolutionary sequence information based on the position-specific scoring matrices generated using the PSI-BLAST tool. A training dataset of 607 proteins used in the NN algorithm was obtained from the X-ray structures in the PDB and the weights and biases of 20 amino acids for α-helices and β-strands were calculated from the secondary structures and amino-acid sequences of these 607 proteins. The positions of α-helices and β-strands in the amino acid sequence were assigned in a descending order of the α-helix and β-strand weights of the 20 amino acids until the determined numbers of α-helix and β-strand residues converged to those estimated from the VUVCD analysis. Next, the numbers of α-helix and β-strand segments estimated from the VUVCD analysis were introduced in NN calculation until the predicted numbers of segments converged to those obtained from VUVCD estimation. If the predicted numbers of residues and segments for α-helices and β-strands did not converge to the VUVCD estimates, the sequence alignment that minimized the difference between the two estimates was taken as the final value. The turns and unordered structures estimated using SELCON3 were classified as “other structures” in the VUVCD-NN method. The predictive accuracy of this method for the positions of α-helix and β-strand segments was 74.9% for the 30 reference soluble proteins (14).

The predictive accuracy obtained from the randomization protocol is around 36.8% (21). Further, when we use only NN method, the accuracy was 70.9% and this accuracy finally improved to 74.9% when the method was combined with the experimental data (14).

The method has been used for the structural analysis of unknown proteins in the native and other states so far (22, 23).

### Purification of GST-Fused NCYM Protein in Bacteria

The open reading frame of NCYM was inserted into the pGEX-6p-1 plasmid so that the GST tag was attached to the N-terminus of NCYM. The plasmid was transformed to BL21 (DE3) cells, which were then grown at 30°C in Luria broth medium supplemented with ampicillin at a concentration of 0.1 mg/mL. At OD = 1.0, protein expression was induced by adding isopropyl-β-D-thiogalactopyranoside at a concentration of 1 mM, followed by 3 h of incubation.

After harvest, the cell pellets were lysed by sonication in phosphate-buffered saline supplement with a protease inhibitor cocktail cOmplete (Roche, Mannheim, Germany). The lysate was subjected to ultracentrifugation and its supernatant was applied to a GSTrap FF column (GE Healthcare, Little Chalfont, UK), which was equilibrated with phosphate-buffered saline. NCYM attached to the GST-tag was purified using elution buffer containing 50 mM Tris-HCl (pH 8.0) and 10 mM reduced glutathione, and the eluate was stored at 4°C. When NCYM was purified without the GST-tag, the column described above was detached from the system and PreScission Protease (GE Healthcare) was added, followed by incubation for 17–18 h at 4°C. After reattaching the column to the system, buffer containing 50 mM Tris-HCl (pH 8.0), 100 mM NaCl, 1 mM EDTA, and 1 mM DTT was used to elute NCYM. Finally, GST-tagged molecules attached to the column were eluted with elution buffer. Using the Bradford method (bovine serum albumin was used as a standard), the yields were determined to be 17.5 and 3.8 mg/L culture for NCYM with and without the GST-tag, respectively.

### Analyses of Single-Nucleotide Polymorphisms in the NCYM Gene

We analyzed single-nucleotide polymorphisms (SNPs) in *NCYM* using the Japanese Multi Omics Reference Panel (jMorp, https://jmorp.megabank.tohoku.ac.jp/202102/variants).

### Cell Culture and Transfection

The human neuroblastoma cell line SH-SY5Y was maintained in DMEM supplemented with 10% fetal bovine serum, 50 U/mL penicillin, and 50 μg/mL streptomycin. The human neuroblastoma cell line IMR32 was maintained in RPMI-1640 medium supplemented with 10% fetal bovine serum, 50 U/mL penicillin, and 50 μg/mL streptomycin.

Plasmid transfections were performed using Lipofectamine 3000 transfection reagent (Invitrogen, Carlsbad, CA, USA) according to the manufacturer’s instructions. At 24 h after transfection, we prepared total RNA for quantitative real-time RT-PCR. At 24 or 72 h after transfection, we prepared cell lysates for western blotting.

### Subcellular Fractionation

To prepare nuclear and cytoplasmic extracts, the cells were lysed in 10 mM Tris-HCl (pH 8.0), 1 mM EDTA, 0.5% Nonidet P-40 (Nacalai Tesque, Kyoto, Japan), and cOmplete™ Protease Inhibitor Cocktail Tablets and centrifuged at 17,800 ×*g* for 10 min to collect the soluble fractions, which were referred to as cytosolic extracts. Insoluble materials were washed with lysis buffer and further dissolved in RIPA buffer to collect the nuclear extracts.

### Western Blotting

Cells were lysed with RIPA buffer, Benzonase (Millipore, Billerica, MA, USA), and MgCl_2_ at final concentrations of 25 U/μL and 2 mM, respectively, incubated at 37°C for 1 h, and centrifuged at 10,000 × *g* for 10 min at 4°C, after which the supernatant was collected. The supernatant was denatured in SDS sample buffer with or without 2-mercaptoethanol (reducing or non-reducing, respectively). Cell proteins were resolved by SDS-PAGE before being electroblotted onto polyvinylidene fluoride membranes. We incubated the membranes with the following primary antibodies for 60 min: anti-NCYM [1:1000 dilution (1)], anti-MYCN antibody (1:1000 dilution; Cell Signaling Technology, Danvers, MA, USA), anti-Lamin B (1:1000 dilution; Millipore), anti-α-tubulin (1:1000 dilution; Cell Signaling Technology), anti-HA (1:1000 dilution; Cell Signaling Technology), and anti-actin (1:1000 dilution; Wako, Osaka, Japan). The membranes were then incubated with horseradish peroxidase-conjugated secondary antibody (anti-rabbit IgG at 1:5000 dilution or anti-mouse IgG at 1:5000 dilution; both from Cell Signaling Technology), and the bound proteins were visualized using a chemiluminescence-based detection kit (ImmunoStar Zeta, Wako; ImmunoStar LD, Wako).

### RNA Isolation and Quantitative Real-Time RT-PCR

The total RNA from plasmid-transfected SH-SY5Y cells was prepared using an RNeasy Mini kit (Qiagen, Hilden, Germany) following the manufacturer’s instructions. cDNA was synthesized using SuperScript II with random primers (Invitrogen). Quantitative real-time RT-PCR (qRT-PCR) using a StepOnePlus™ Real-Time PCR System (Thermo Fisher Scientific, Waltham, MA, USA) was performed with SYBR green PCR. The following primer sets were used: *MYCN*, 5′-TCCATGACAGCGCTAAACGTT-3′, and 5′-GGAACACACACAAGGTGACTTCAAC-3′. *β-actin* expression was quantified using the TaqMan real-time PCR assay. The mRNA levels of *MYCN* gene were standardized using that of *β-actin*.

### Vector Construction

Plasmid vectors were synthesized by GenScript Japan (Tokyo, Japan) as follows. Plasmid vectors encoding the HA-NCYM ORF (WT) and amino acid mutants of HA-NCYM ORF (E7G(A20G), N52S(A155G), G59R(G175A), L63P(T188C), Y66S(A197C), L70V(C208G), V71D(T 212A), G78E(G233A), D90N(G268A), and E98G(A293G)) were synthesized using the restriction enzymes KpnI and BamHI with pcDNA3. 1-N-HA is a vector with a CMV promoter for expressing proteins with an HA tag at the N-terminus. The start codon of the ORF of NCYM was deleted.

## Results

### VUVCD Analyses Revealed the Secondary Structure of the NCYM Protein

Significant differences in the spectra were observed in the wavelength range of 190–240 nm between the tagged proteins and tag only solution (**Figure 1C**). These differences arise from the spectra of NCYM. The secondary structure contents and segments of the isolated SUMO tag were analyzed using the VUVCD spectra and SELCON3 program (15, 19). The isolated SUMO tag (129 residues) contained 14.2% α-helices and 29.2% β-strands in the hydrogenated state and 13.9% α-helices and 28.7% β-strands in the perdeuterated state (**Table 1**). The atomic structure (PDB ID: 3PGE) of tag fragment (80 residues) from X-ray crystallography showed that this fragment contains 14% helices and 32% sheets. The lengths of the tags in the X-ray and VUVCD methods differed but the secondary structure contents estimated by VUVCD agreed well with those of the crystal structure. The SUMO-tagged NCYM (238 residues) in the hydrogenated state was found to contain 17.1% α-helices and 27.2% β-strands (**Table 1**), indicating that NCYM forms the characteristic secondary structures when tagged with SUMO. To investigate the disordered nature of *de novo* evolved proteins, we synthesized perdeuterated NCYM with a SUMO tag and the isolated SUMO tag using an *in vitro* cell-free system (**Figure 1B**). Small differences in the spectra were observed in the wavelength range of 210–220 nm between perdeuterated and hydrogenated NCYM (**Figure 1C**). The secondary structure analysis showed that perdeuterated SUMO-tagged NCYM contains 16.3% α-helices and 27.0% β-strands (**Table 1**). The secondary structure contents between perdeuterated and hydrogenated NCYM were identified within the calculation error range of SELCON 3 program, but the differences between both spectra around 220 nm affect the helical contents because the CD intensity at 222 nm is highly sensitive to the amount of helical structure (13).

**Table 1:** Secondary structure content of the NCYM samples used for the VUVCD measurements.

	N_res_	H(r)	H(d)	S(r)	S(d)	N_helix_	N_strand_
H_Tag_NCYM	238	6.7	10.4	15.9	11.3	6	13
D_Tag_NCYM	238	6.5	9.8	15.9	11.1	6	13
H_Tag	129	5.4	8.8	16.6	12.6	3	8
D_Tag	129	4.9	9.0	16.3	12.4	3	8

N_res_ denotes the number of residues contained in the samples. H(r), H(d), S(r), and S(d) are the fraction (%) of ordered α-helix, disordered α-helix, ordered β-strand, and disordered β-strand, respectively. N_helix_ and N_strand_ show the number of α-helix and β-strand (sum of the ordered and disordered structures) contained in the corresponding samples, respectively.

### Prediction of the Secondary Structure of NCYM at the Single Amino-Acid Level

To predict the secondary structure of NCYM at the amino-acid sequence level, we used the sequence-based prediction method (PSIPRED, JPred4, trRosetta, and RaptorX), which can estimate the secondary structure only from the amino-acid sequence of the target protein (24–27). The predicted results and the estimated secondary structure contents are shown in **Figure S1**. Evidently, these results had large variations in the region of the NCYM. Furthermore, the secondary structure contents from the VUVCD results (**Table 1**) are different to those obtained from these sequence-based prediction methods. This indicates that the prediction of the secondary structure of NCYM would not be adequate for the current algorithms, probably due to specificities of amino-acid sequence of *de novo* evolved protein. Hence, from the perspective of the secondary structure contents, we used the experimental data obtained from the VUVCD analysis to predict the secondary structures of NCYM at the amino-acid sequence level.

We predicted the positions of the secondary structures in SUMO-tagged NCYM and the isolated SUMO tag at the amino acid sequence level (**Figures 1D** and **S2**) using the neural network system combined with the contents and segments of secondary structures obtained using the VUVCD analysis (**Table 1**) (14). The predicted sequence of the secondary structure of the tag was consistent with that of X-ray crystallography with 75% accuracy (data not shown), which is the same as the average performance for the 30 reference proteins (15). Comparisons between the secondary structure sequences of SUMO-tagged NCYM and the isolated SUMO tag revealed that NCYM contains seven β-strands and four α-helices (**Figure 1D**). Three of the four α-helices were localized in the central region of NCYM. In addition, the protein database UniProt revealed the presence of compositional bias to Arg/Lys-rich (basic) at the C-terminal (68–109 aa) of NCYM (UniProt KB-P40205 NCYM Human, **Figure 1D**). According to the amino acid sequence only, NCYM was previously predicted to be a basic helix-loop-helix protein (28) (**Figure S3**), but the predicted regions of the helix in the present study differed from those in the previous report. This is likely because we considered the secondary structure contents experimentally obtained from the VUVCD method (**Table 1**). The sequences of secondary structures of hydrogenated and perdeuterated NCYM proteins were identical to each other but showed slight differences in aspartic acid (D90) (other structure in the perdeuterated protein and α-helical structure in the hydrogenated structure), as shown in **Figure 1D**. This indicates that D90 might perturb the conformation of the NCYM. Note that “other structure” comprises all secondary structures other than α-helix and β-strand. Considering the experimental and analytical errors, the differences in the secondary structures of SUMO-tagged NCYM between hydrogenated and perdeuterated forms including D90 assignment are expected to be within the error range. Nevertheless, as aspartate often plays a major role in protein function, as in the catalytic triad, the secondary structural change at D90 from α-helix (hydrogenated state) to other structures (perdeuterated state) suggests that this residue is more prone to differences in local physicochemical environments (hydrogenated *vs.* perdeuterated) and is thus involved in the molecular stability of NCYM and its function. Therefore, it is worth investigating the possible role of D90 in the interaction with MYCN or GSK3β, especially focusing on the negative charge of D90. We chose asparagine for generating NCYM D90 mutant because it resembles aspartic acid the most, with a difference only in one chemical group change which made the residue polar instead of charged. For this purpose, the D90N mutant was generated and its effect on NCYM function was studied, although D90N is yet to be detected as a naturally occurring mutation in humans.

### NCYM Forms Oligomers

Because the structurally characterized *de novo* evolved protein Bsc4 forms oligomers (12), we examined the oligomer structure formation ability of NCYM. The NCYM protein was synthesized and purified from bacterial cells (**Figure S4A**). The bands of purified NCYM were detected at the predicted sizes of monomers, dimers, trimers, and tetramers *via* non-reducing SDS-PAGE (**Figure S4B**). Next, whole cell extracts of *MYCN*-amplified neuroblastoma cells IMR32 were prepared to detect oligomers of endogenous NCYM (**Figure S4C**). To prevent the oligomers of NCYM from degrading, the samples were prepared without sonication. The addition of benzonase increased the concentration of soluble NCYM protein, and the bands of endogenous NCYM were detected at the predicted sizes of monomers, dimers, trimers, tetramers, and pentamers in a western blot under reducing conditions (**Figure S4C**). To study the subcellular localization of NCYM oligomers, western blotting of the nuclear fraction was performed. Monomeric bands of NCYM were detected in the nucleus, and bands predicted to be dimers, trimers, and tetramers were detected in the cytoplasm (**Figure S4D**).

### Non-Synonymous SNPs in NCYM Are Enriched in the Domain Structure

**Figure 2:**
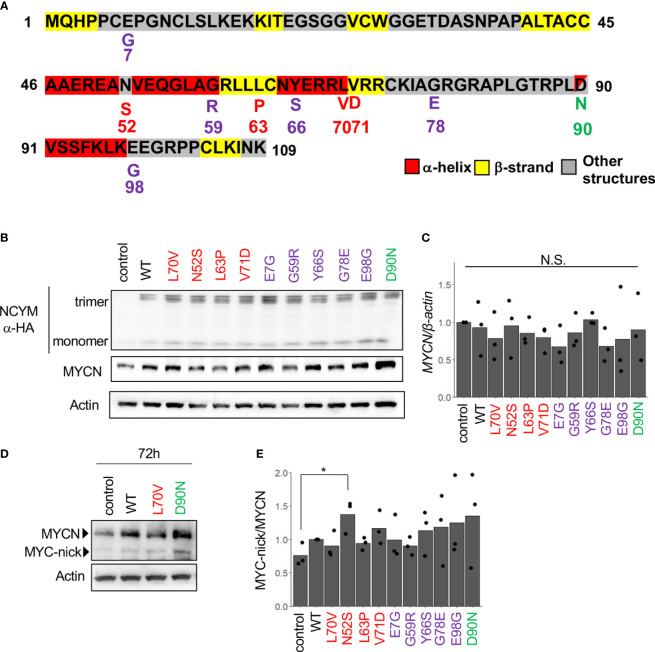
MYCN stabilization and Myc-nick production by each NCYM variant. **(A)** NCYM amino acid sequences. Purple, variants found in chimpanzees; red, variants found in humans; green, D90N, a mutant of NCYM in which the 90th aspartic acid is substituted with asparagine. **(B)** Western blotting of HA-NCYM, MYCN proteins in NCYM SNP plasmid-transfected SH-SY5Y cells. At 24 h after transfection, the cells were subjected to western blotting. Actin was used as a loading control. Control: empty vector. **(C)** Quantitative real-time RT-PCR analyses of *MYCN* in NCYM SNP plasmid-transfected SH-SY5Y cells. At 24 h after transfection, mRNA expression levels were measured by real-time RT-PCR with *β-actin* as an internal control. N.S., not significant. Data were analyzed using Student’s *t* test (comparison with control). **(D)** Western blotting of MYCN and Myc-nick proteins in NCYM SNP plasmid-transfected SH-SY5Y cells. At 72 h after transfection, cells were subjected to western blotting. Actin was used as a loading control. **(E)** Quantification of western blotting analysis of NCYM SNP plasmid-transfected SH-SY5Y cells. At 72 h after transfection, the cells were subjected to western blotting. Myc-nick level was normalized to MYCN level. Data are shown as plots and means of three independent experiments. *p < 0.05. Data were analyzed using Student’s *t* test (comparison with control).

Next, we conducted a search for SNPs of NCYM in the human population using the Japanese Multi Omics Reference Panel (jMorp, **Supplementary Table 1**). All four non-synonymous SNPs (N52S, L63P, L70V, and V71D) in humans and two of five non-synonymous substitutions in chimpanzee (G59R and Y66S) were found to be accumulated in the central domain structure of the NCYM (**Figure 2A**). The frequencies of these SNPs differed among people of different ethnicities, with the highest proportion observed for L70V. Substitution of the 35th serine, a phosphorylation site (Japan Proteome Standard Repository/Database, ID: PSM204_1_28892), of NCYM to cysteine (S35C) was detected at a frequency of 0.0001 in African people. A frameshift mutation causing a deletion in the NCYM (I19N*17) was found in Japanese, African, and Non-Finnish European people, and it causes the protein to have similar amino acid sequences as the ancestral form of NCYM (**Table 2**). A previous study reported that NCYM stabilizes the MYCN protein (1) and promotes Myc-nick production (3). To investigate the effect of amino acid substitutions on the function of NCYM, variants of NCYM including the D90N mutant were overexpressed in the neuroblastoma cell line SH-SY5Y. Western blotting revealed that the expression of MYCN increased upon overexpression of the NCYM variant of D90N (**Figure 2B**). The real-time RT-PCR showed no change in the mRNA expression level of *MYCN* (**Figure 2C**). We detected Myc-nick production at 72 h (**Figure 2D**), and N52S mutation of NCYM significantly increased Myc-nick production (**Figure 2E**).

**Table 2:** Frequency of NCYM SNPs.

ref SNP	Variant	Frequency						Remark
		AFR	NFE	AMR	ASJ	EAS	JPN	
rs181162426	N52S	0.0002	–	–	–	0.0263	0.0022	High frequency (>0.01)
rs190044705	L63P	–	–	–	–	0.0006	0.0237	High frequency (>0.01)
rs11886063	L70V	0.9986	0.9958	1	1	1	1	High frequency (>0.01)
rs75731159	V71D	–	0.0007	–	–	0.0148	0.0383	High frequency (>0.01)
rs919133132	I19N*17	0.0002	0.0004	–	–	–	0.0001	Ancestral variant
Rs973265238	S35C	0.0001	–	–	–	–	–	Phosphorylation site

JPN, Japanese; AFR, African; AMR, American; ASJ, Ashkenazi Jewish; EAS, East Asia; NFE, Non-Finnish European. - indicates not detected.

## Discussion

In this study, we revealed the secondary structure of NCYM and showed that α-helices and β-strands were enriched in the central region (amino acids 40–73) of NCYM, which emerged in Homininae by two frameshifts in the ORF (1, 5). Furthermore, it was found that the D90N mutation activated NCYM-mediated MYCN stabilization. Within the ORF of NCYM, all four nonsynonymous SNPs were in the central region, three of which showed high frequency in East Asians and N52S promoted the NCYM function to produce Myc-nick. Therefore, the central region of NCYM appears to be a functional domain responsible for NCYM-mediated Myc-nick production.

Although the D90N mutation is not one of the naturally occurring mutations, we found that D90N increases MYCN stabilization, suggesting that the negative charge in D90 has an inhibitory effect on the binding of NCYM to MYCN or GSK3β. Thus, the use of both hydrogenated and perdeuterated NCYM proteins enabled to identify a residue that significantly modulates NCYM function, raising a possibility that protein deuteration might be useful for studying the molecular mechanism of protein activity. Moreover, further systematic biochemical studies using different mutations at D90 would be beneficial to decipher the effects of volume, polarity, and hydrophobicity of the residue at this site on NCYM function.

In this study, the secondary structure of SUMO-tagged NCYM was analyzed. In the intrinsically disordered regions in proteins, their transiently folded and disordered states are typically in equilibrium with each other, and the interaction with other molecules shifts this equilibrium toward the former (29). Although the effect of non-covalent interactions with other proteins on protein structure may be different from that of the covalent binding of the tag, it is possible that the SUMO tag stabilizes NCYM conformation, thus facilitating the folding of some regions in NCYM that would otherwise be disordered when isolated. In this case, it indicates that the regions of NCYM where the folded secondary structure was assigned are susceptible to a slight change in local physicochemical environments caused by binding of the SUMO tag, suggesting that these regions contribute to the molecular stability of NCYM. Therefore, in order to better understand the structural properties of NCYM, the folded state of NCYM with SUMO tag should be compared with the structure of the isolated NCYM in the near future.

The western blotting analysis revealed that NCYM exists as oligomers in addition to monomers, and NCYM oligomers were detected even under reducing conditions. This suggests that the interaction between monomers in the NCYM oligomers is strong enough not to be disrupted easily by the thermal energy. Therefore, it is possible that the secondary structure of NCYM changes as it forms oligomers, especially in the binding interface between monomers. The fact that NCYM oligomers are detected in the fraction of the cytoplasm while NCYM monomers are detected in the fraction of the nucleus implies that subcellular localization of these molecular species might be an important factor for NCYM function, which requires structural characterization of NCYM oligomers and monomers.

The present study also revealed the first function-structure relationships of *de novo* evolved proteins. *De novo* evolved proteins, by definition, lack homology to known proteins or known functional domains and show high flexibility, as observed in intrinsically disordered proteins (11). These features have impeded the functional and structural characterization of *de novo* evolved proteins. The present study suggests that synchrotron-radiation VUVCD with perdeuterated proteins is a promising strategy for identifying functional domain structures of *de novo* evolved proteins. SNPs in populations are also useful for functional characterization of *de novo* evolved proteins because they occasionally include amino acids that are essential for protein functions.

The existence of nonsynonymous SNP that affects NCYM-mediated Myc-nick production supports that *NCYM* is a bona fide protein-coding gene (1, 5), rather than a long noncoding RNA. Nonsynonymous SNPs at the phosphorylation site of NCYM (S35C) further support that *NCYM* encodes proteins. In addition to noncoding variants of *NCYM*/*MYCNOS* (NR_110230, transcript variant 1 in the National Center for Biotechnology Information database) (30, 31), the transcript variants of coding *NCYM* (NR_161162 and NR_161163, transcript variants 2 and 3) have been proposed to function as long noncoding RNAs that enhance MYCN expression (32, 33) and promote metastasis (32), as reported for protein function (1). Two contradictory molecular mechanisms have been proposed for MYCN induction as mediated by noncoding *NCYM* transcripts (32, 33). One study reported that *NCYM* noncoding RNA stimulates the upstream promoter of *MYCN via* the recruitment of CTCF (32), and the other showed that *NCYM* noncoding RNA suppressed the upstream promoter and stimulated the internal promoter activity of *MYCN*, resulting in efficient translation of MYCN (33). Under our experimental conditions, we did not detect *MYCN* mRNA induction after transduction of wild-type NCYM, and no significant difference in *MYCN* mRNA levels was observed in cells that overexpressed NCYM variants. Further studies are required to examine whether these variants affect the noncoding functions of NCYM.

Orthologous transcripts of *NCYM* are widely detected in mammals (5), but the ORF emerged in Homininae during evolution (1, 5). Similar to other *de novo* genes (34), we detected ORF-disrupting SNPs which caused the *NCYM* gene resemble the ancestral-type sequence. Furthermore, one NCYM SNP mutant found in East Asians promoted Myc-nick production. In addition to neuroblastomas, high expression of *MYCN* or *NCYM* is associated with poor outcomes in hepatocellular carcinomas (35) or cholangiocarcinomas (5), respectively. Therefore, the roles of NCYM SNPs in the development of these tumors, frequently found in East Asians, should be further studied.

## Data Availability Statement

The original contributions presented in the study are included in the article/**Supplementary Material**. Further inquiries can be directed to the corresponding authors.

## Author Contributions

TM, KN, KM, TT, and YS performed the experiments, acquired, and analyzed the data. TM, KN, KM, TS, TT, and YS wrote the manuscript. KN, TS, TT, and YS acquired funds. TT and YS designed and supervised the study. All authors contributed to the article and approved the submitted version.

## Funding

This work was partially supported by the Interstellar Initiative (grant number 18jm0610006h0001 to YS) from the Japan Agency for Medical Research and Development, Grant-in-Aid for Scientific Research (C) (JSPS Kakenhi Grant No. 18K08162 and No. 21K08610 to YS), Grant-in-Aid for Scientific Research (C) (JSPS Kakenhi Grant 20K09338 to TS) from the Japan Society for the Promotion of Science, Innovative Medicine CHIBA Doctoral WISE Program (to KN) from Chiba University, Kawano Masanori Memorial Public Interest Incorporated Foundation for Promotion of Pediatrics (to YS).

## Conflict of Interest

The authors declare that the research was conducted in the absence of any commercial or financial relationships that could be construed as a potential conflict of interest.

## Publisher’s Note

All claims expressed in this article are solely those of the authors and do not necessarily represent those of their affiliated organizations, or those of the publisher, the editors and the reviewers. Any product that may be evaluated in this article, or claim that may be made by its manufacturer, is not guaranteed or endorsed by the publisher.

## Supplementary Material

Supplementary Figure 1 Secondary structures of SUMO-tagged NCYM using the sequence-based prediction methods. **(A)** From top to bottom, positions of the SUMO-tagged NCYM predicted using PSIPRED, JPred4, trRosetta, and RaptorX are shown. α-Helix, β-strand, and other structures are shown in red, yellow, and gray, respectively. **(B)** Contents of α-helix, β-strand, and other structures estimated using the prediction methods used in **(A)**. N_helix_ and N_strand_ denote the number of α-helix and β-strand, respectively.

Supplementary Figure 2 Secondary structure of hydrogenated SUMO-tagged NCYM (H_Tag_NCYM), perdeuterated SUMO-tagged NCYM (D_Tag_NCYM), hydrogenated SUMO tag (H_Tag), and perdeuterated SUMO tag (D_Tag) predicted by the neural network. Note that in the region I119–P133 of H_Tag_NCYM, there are two β-strands, that is, I119–G128 and G129–P133. The residues belonging to the disordered β-strand, which are assigned by the neural network, correspond to the edges of the β-strands (I119, G128, G129, and P133 in this case). The fraction of the ordered and disordered β-strand is listed in **Table 1**.

Supplementary Figure 3 Comparison of the structure of NCYM between the present study and previous report.

Supplementary Figure 4 NCYM forms oligomers. **(A)** Purification of NCYM. **(B)** Western blotting showing the monomer, dimer, trimer, and tetramer structures of purified NCYM in non-reducing conditions, but the protein did not form an oligomer under reducing conditions. **(C)** Western blots showing monomer, dimer, trimer, tetramer, and pentamer structures of NCYM from IMR32 cells. **(D)** Western blotting of nuclear and cytoplasmic fractions from IMR32 cells. Monomers and oligomers of NCYM were found in the nucleus cytoplasm, respectively.

Supplementary Table 1 Single-nucleotide polymorphisms (SNPs) in *NCYM*.
