# Supplementary figures and images for: Secondary Structure of Human De Novo Evolved Gene Product NCYM Analyzed by Vacuum-Ultraviolet Circular Dichroism
Source: Front Oncol. 2021 Aug 23;11:688852. doi: 10.3389/fonc.2021.688852 (PMC8420857; doi:10.3389/fonc.2021.688852)

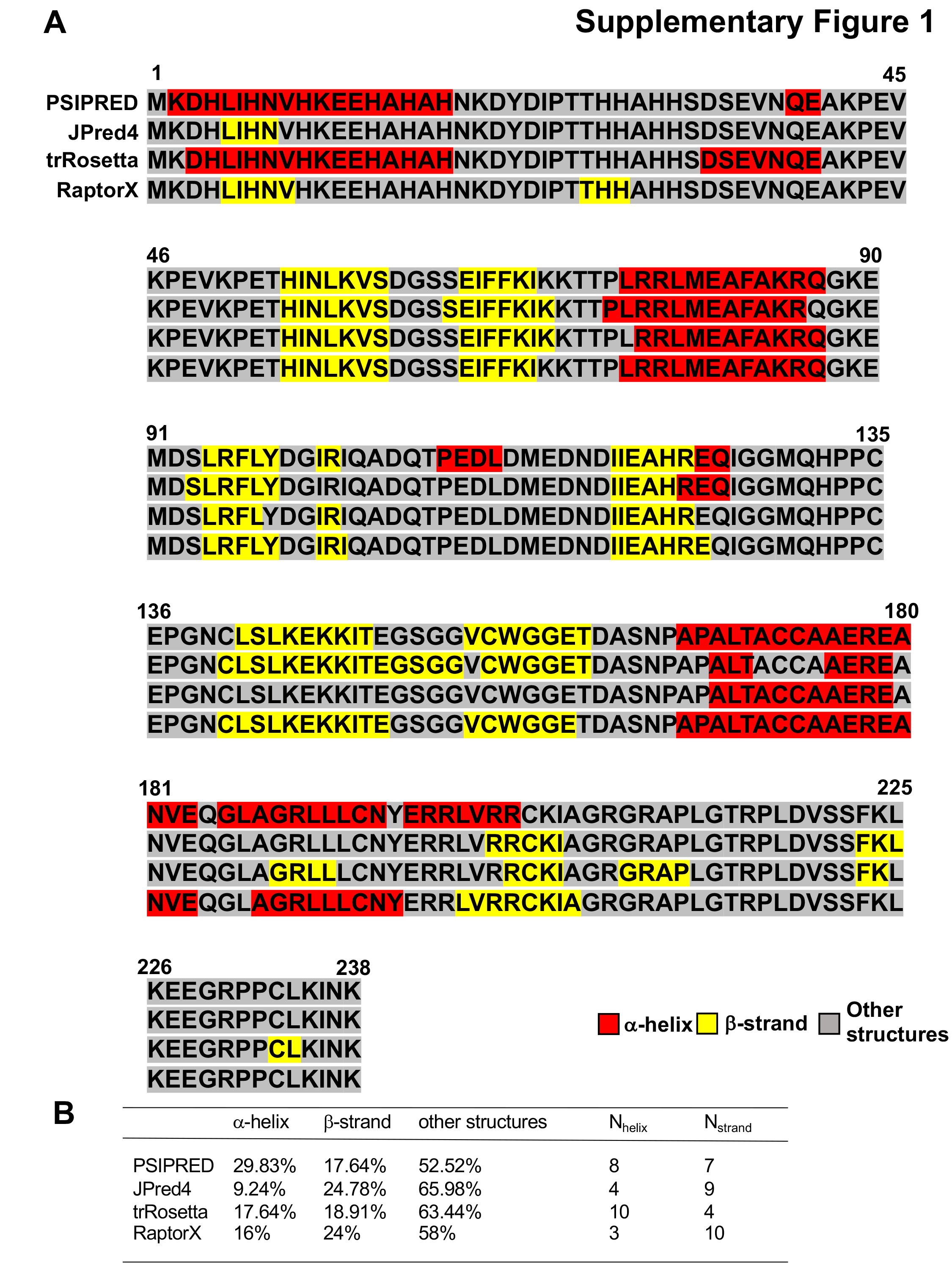

Supplement: Supplementary Figure 1 — Secondary structures of SUMO-tagged NCYM using the sequence-based prediction methods. (A) From top to bottom, positions of the SUMO-tagged NCYM predicted using PSIPRED, JPred4, trRosetta, and RaptorX are shown. α-Helix, β-strand, and other structures are shown in red, yellow, and gray, respectively. (B) Contents of α-helix, β-strand, and other structures estimated using the prediction methods used in (A). Nhelix and Nstrand denote the number of α-helix and β-strand, respectively. [file Image_1.tif]

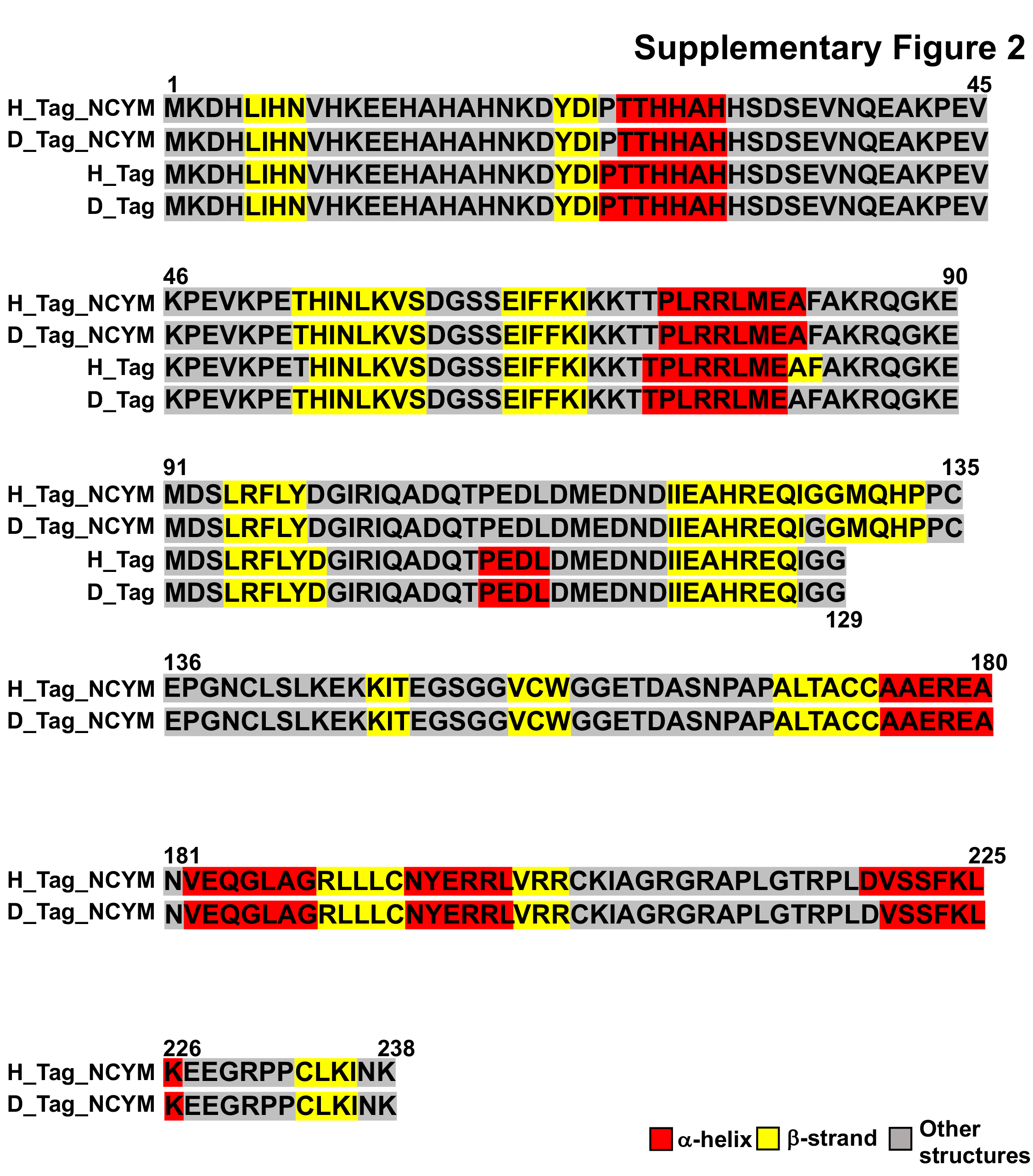

Supplement: Supplementary Figure 2 — Secondary structure of hydrogenated SUMO-tagged NCYM (H_Tag_NCYM), perdeuterated SUMO-tagged NCYM (D_Tag_NCYM), hydrogenated SUMO tag (H_Tag), and perdeuterated SUMO tag (D_Tag) predicted by the neural network. Note that in the region I119–P133 of H_Tag_NCYM, there are two β-strands, that is, I119–G128 and G129–P133. The residues belonging to the disordered β-strand, which are assigned by the neural network, correspond to the edges of the β-strands (I119, G128, G129, and P133 in this case). The fraction of the ordered and disordered β-strand is listed in Table 1. [file Image_2.tif]

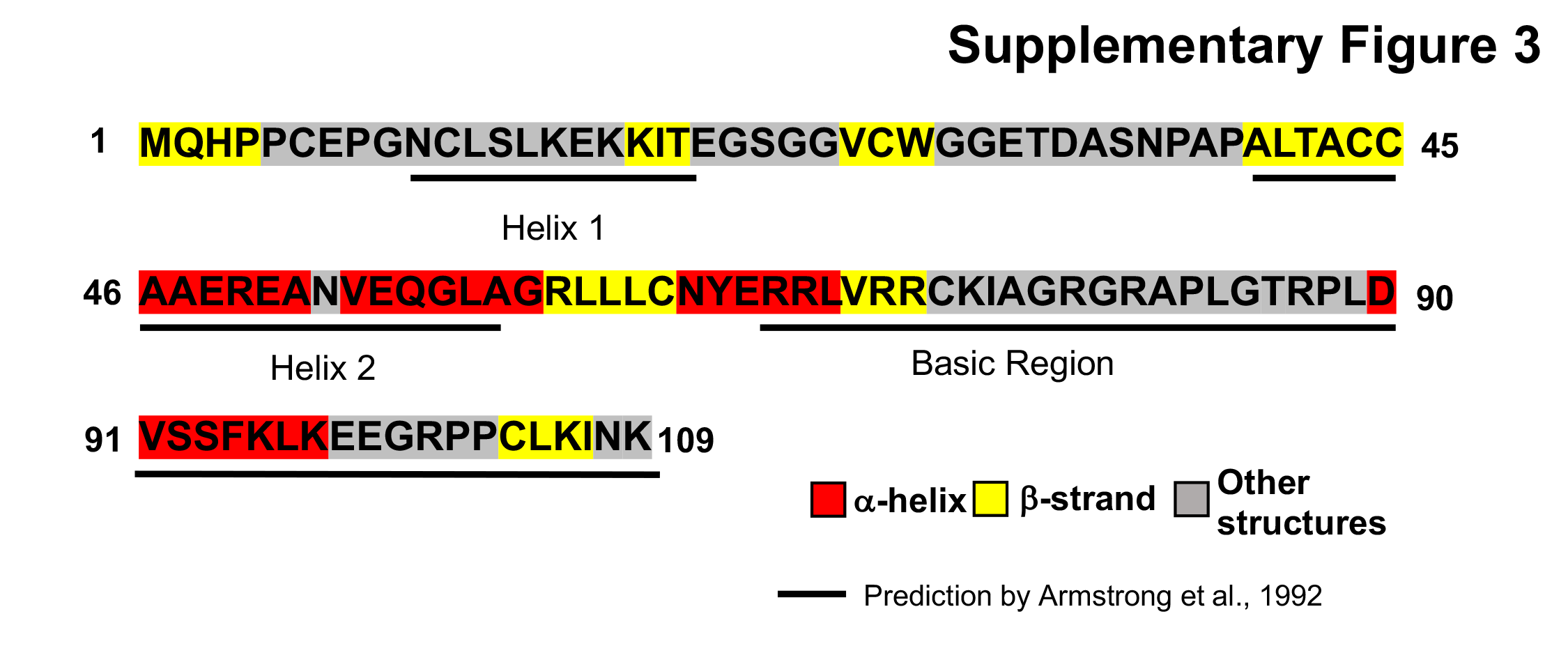

Supplement: Supplementary Figure 3 — Comparison of the structure of NCYM between the present study and previous report. [file Image_3.tif]

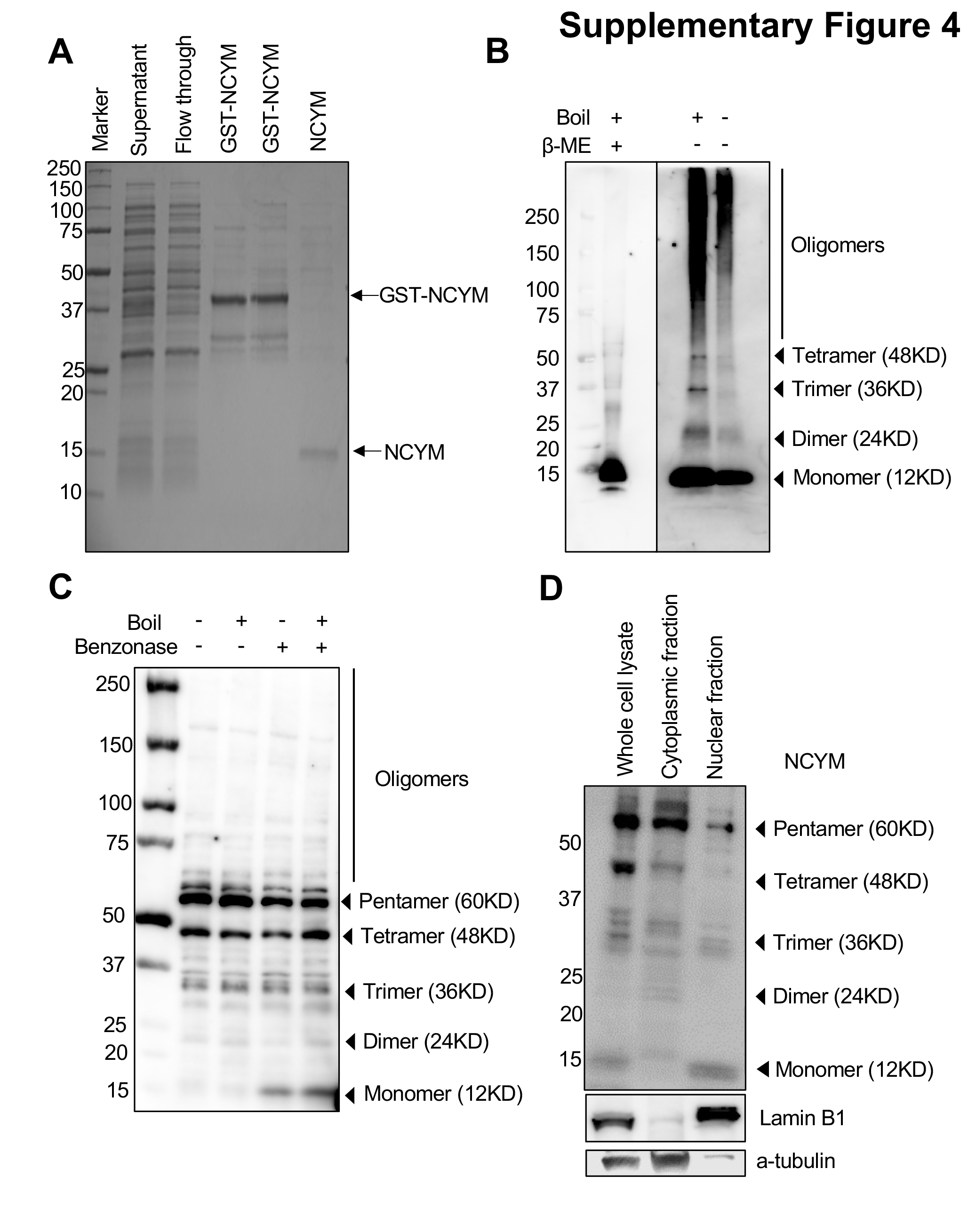

Supplement: Supplementary Figure 4 — NCYM forms oligomers. (A) Purification of NCYM. (B) Western blotting showing the monomer, dimer, trimer, and tetramer structures of purified NCYM in non-reducing conditions, but the protein did not form an oligomer under reducing conditions. (C) Western blots showing monomer, dimer, trimer, tetramer, and pentamer structures of NCYM from IMR32 cells. (D) Western blotting of nuclear and cytoplasmic fractions from IMR32 cells. Monomers and oligomers of NCYM were found in the nucleus cytoplasm, respectively. [file Image_4.tif]
